# Impact of rich cultural tourism experience on tourist satisfactions and behavioral intentions toward Ningxia’s cultural heritage: Moderation role of perceived cultural distance

**DOI:** 10.1371/journal.pone.0336220

**Published:** 2025-11-06

**Authors:** Lei Qin, Hongmei Zhang

**Affiliations:** 1 Business School, North Minzu University, Yinchuan, China; 2 Management School, North Minzu University, Yinchuan, China; Macau University of Science and Technology, MACAO

## Abstract

Ningxia is a renowned cultural tourism destination with rich heritage and natural landscapes. Current research, however, remains unclear on how multi-dimensional cultural experiences influence tourist behavior-particularly the mediating role of cultural identity (CI) and the moderating effect of perceived cultural distance (PCD). To address this gap, this study expands the Cognitive-Affective-Behavioral model and examines how Rich Cultural Tourism Experiences (RCTE) affect satisfaction and behavioral intentions, using network semantic analysis (14,163 texts) and PLS-SEM (547 questionnaires). Five RCTE dimensions are identified: historical, modern, folk, spiritual, and ecological. Results show that RCTE boosts satisfaction, which in turn enhances behavioral intentions. Among the dimensions, spiritual, historical, and ecological experiences exert the strongest effects on CI, with path coefficients of 0.247, 0.244, and 0.200 respectively. CI serves as a key mediator, while PCD weakens RCTE’s impact on CI-but not on satisfaction.

## Introduction

Ningxia Hui Autonomous Region, located in northwestern China, has become a popular tourist destination for both domestic and international visitors due to its rich cultural heritage and diverse ethnic cultures [1]. During the summer of 2024 (June to August), Ningxia received 18.3462 million tourists, representing a year-on-year growth of 20.93%. During this period, total tourism revenue reached 18.613 billion yuan, a year-on-year increase of 21.62%. Beyond cultural heritage, government policies significantly contributed to this growth [2]. For instance, the local government introduced a series of preferential policies like distributing travel vouchers and offering entrance ticket discounts during peak seasons. Moreover, “tourism + sports” and “tourism + performance” models, such as hosting music festivals and sports events, further spurred tourist visits and spending. These achievements were primarily driven by tourists engaging in activities related to cultural heritage, such as sightseeing at scenic spots, exploring museums, and experiencing intangible cultural heritage [2]. This highlights the significant role that cultural heritage tourism has played in the growth of the region’s tourism industry [3].

Ningxia was selected as the study context for its unique cultural representativeness and practical significance. As a hub of the local culture and ancient Silk Road heritage, it integrates ethnic traditions, historical sites, and natural landscapes, attracting tourists from diverse cultural backgrounds [2]. Moreover, Ningxia’s policy focus on integrating intangible heritage with modern infrastructure provides a real-world setting to explore cultural tourism dynamics. Ningxia integrates intangible heritage with modern infrastructure via coordinated initiatives-led by its Culture and Tourism Department and local marketing alliances (e.g., Tiktok recommendation officer)-and even launched a “Digital Cultural Tourism Platform” to measure campaign impact with social media analytics [4]. Yet existing literature has a critical gap: it rarely explores how Ningxia’s multi-dimensional cultural tourism experiences influence tourist outcomes, nor the mechanisms linking them to cultural identity, satisfaction, or how perceived cultural distance interacts-hindering targeted strategies, making this study necessary.

Ningxia’s cultural heritage includes ancient historical sites, vibrant ethnic customs, and unique natural landscapes. These attractions draw numerous tourists from diverse cultural backgrounds [4]. Its diverse cultural offerings serve as a unique platform for cross-cultural exchanges. Thus, understanding how tourists from different backgrounds perceive and engage with these experiences is vital. However, existing research has a critical gap. It rarely clarifies how perceived cultural distance-an individual’s subjective judgment of differences between their own culture and the destination’s (e.g., language, customs, values)-shapes tourists’ cultural identity and satisfaction in Ningxia’s context [5]. Greater cultural differences may spark novelty or create understanding barriers [5], and Perceived Cultural Distance (PCD) moderates the “Rich Cultural Tourism Experience (RCTE)-cultural identity” and “RCTE-satisfaction” paths inconsistently. On the RCTE-cultural identity path, PCD negatively moderates. RCTE fosters cultural identity by building a “sense of shared identity” (tourists’ perception of connections with the destination’s values/symbols). Yet larger PCD widens cognitive-emotional gaps, hindering alignment-even with in-depth RCTE, tourists struggle to feel “connected” weakening identity formation. To turn this positive, destinations can add “cultural bridges” like guided ritual explanations or folk craft workshops to resolve confusion and build affinity. On the RCTE-satisfaction path, PCD shows no significant moderating effect. This is because tourists’ satisfaction with RCTE mainly depends on the quality of the experience itself-such as the authenticity of cultural displays, the professionalism of service staff, or the completeness of activity arrangements-rather than the degree of cultural alignment with the destination. Even with large PCD, as long as RCTE meets tourists’ expectations for “richness” and “authenticity” it can still drive high satisfaction; poor experience quality, by contrast, will reduce satisfaction regardless of PCD levels.

Rich cultural tourism refers to the process in which tourists engage in in-depth exploration and understanding of a destination’s culture by participating in local cultural activities, learning about historical backgrounds, and experiencing traditional customs [6]. Compared with cultural tourism, rich cultural tourism represents a comprehensive and immersive engagement with a destination’s culture, where tourists go beyond superficial encounters and actively participate in the local cultural fabric. This immersive cultural interaction not only strengthens tourists’ sense of cultural identity but also alleviates discomfort caused by perceived cultural distance, thereby enhancing satisfaction [7]. As an effective way to increase tourist satisfaction, rich cultural tourism experiences have a positive impact on reinforcing tourists’ behavioral intentions and loyalty.

Moreover, a rich cultural tourism experience positively influences cultural identity by deepening tourists’ connection and understanding of the destination’s culture [7]. Cultural identity refers to an individual’s sense of belonging to a particular culture, shaped by shared values, traditions, language, and customs that influence their self-perception and connection to a community [8]. When tourists engage in meaningful interactions with local traditions, history, and customs, they develop a stronger sense of cultural identity, feeling more aligned with and integrated into the cultural context of the destination [9]. The study defines “cultural identity” as tourists’ identification with Ningxia’s local culture, primarily focusing on domestic Chinese tourists. This enhanced cultural identity then has a positive impact on both satisfaction and tourists’ behavioral intentions. Tourists who feel a greater sense of cultural identification are more likely to report higher levels of satisfaction, as the cultural experience resonates with their personal values and enhances the overall enjoyment of their trip [10]. Additionally, this sense of cultural belonging fosters stronger behavioral intentions, such as the desire to revisit the destination, recommend it to others, or engage in future cultural tourism activities [11]. Thus, a rich cultural tourism experience not only enriches tourists’ cultural identity but also plays a significant role in improving their overall satisfaction and reinforcing their loyalty and commitment to the destination.

This study makes three distinct contributions to the field of cultural tourism research. First, it constructs a five-dimensional model of rich cultural tourism experience-encompassing historical, modern, folk, spiritual, and ecological dimensions-to reveal how these components differentially influence tourist satisfaction, thereby challenging traditional single-dimensional frameworks. Second, this study extends the Cognitive-Affective-Behavioral framework by unpacking the cognitive component into distinct, context-specific dimensions of rich cultural tourism experiences (historical, modern, folk, spiritual, ecological). Unlike prior research that treats cognitive inputs as a monolith, it identifies differential effects-e.g., spiritual and historical experiences strengthen the cognitive-to-affective (satisfaction) link more profoundly than modern or folk ones. Additionally, it empirically validates the sequential transmission mechanism (cognitive experiences → affective satisfaction → behavioral intentions) in a cultural heritage context, clarifying how specific cognitive elements drive affective responses and subsequent behavioral outcomes, thus refining the framework’s application to tourism research. Finally, by integrating network semantic analysis with PLS-SEM modeling, the study bridges qualitative cultural coding and quantitative structural analysis, offering a novel methodological approach for examining cross-cultural tourism dynamics. These insights provide both theoretical advancements to the Cognitive-Affective-Behavioral model and practical strategies for enhancing cultural heritage management in destinations like Ningxia.

## Literature review and research hypotheses

### Rich cultural tourism experience

Rich cultural tourism in Ningxia offers a rich experience by connecting tourists with its historical, modern, folk, spiritual, and ecological dimensions [12,13]. Historically, tourists can engage with Ningxia’s ancient sites, such as the Western Xia Mausoleum and the Water Cave Gully, gaining insight into the region’s significance in Chinese history [12]. Modern cultural tourism, on the other hand, presents a blend of tradition and innovation, allowing visitors to see how Ningxia integrates its historical heritage with contemporary developments, offering a dynamic view of cultural evolution [13].

In addition, Ningxia’s folk, spiritual traditions, and ecological environment provide rich layers of cultural immersion [4,14,15]. Spiritual cultural experiences in Ningxia heritage tourism encompass the satisfaction derived from connecting with the region’s spiritual sites and traditions [15]. Besides, tourists can learn about traditional myths and practices that have shaped the region’s identity [14]. The ecological aspect, involving the exploration of natural landscapes like the Helan Mountains and the Yellow River, further enriches the experience by highlighting the interconnection between Ningxia’s environment and its cultural practices [4]. Salazar (2012) argues that cultural immersion thrives on narrative-rich traditions, which supports our focus on folk and spiritual elements as core to deep engagement [14]. Nie et al. (2022) illustrate how ecological contexts anchor cultural practices, reinforcing our framework’s integration of natural landscapes [4]. Robinson (2024) emphasizes spiritual experiences as emotional bridges, aligning with our emphasis on their role in holistic cultural immersion [15]. These studies collectively validate the multi-layered structure of our experiential framework.

International parallels enrich these five dimensions [16,17]. Europe mirrors Ningxia’s historical focus through sites like Rome’s Colosseum, while its modern blend (e.g., Amsterdam’s tech-enhanced heritage displays) aligns with Ningxia’s tradition-innovation fusion. The Middle East echoes folk elements via Lebanon’s village festivals, akin to Ningxia’s local customs; its spiritual tourism resonates with Helan Mountain’s significance. Ecologically, Jordan’s Wadi Rum desert tours parallel Ningxia’s desert-river ecosystems, highlighting universal and region-specific traits in “rich cultural tourism”. “Rich cultural tourism” in this context transcends conventional “cultural tourism” (which often focuses on singular heritage sites) by integrating five interconnected dimensions. It emphasizes holistic immersion: not just visiting historical relics, but engaging with modern cultural innovations, folk customs, spiritual traditions, and ecological landscapes as an interconnected whole, creating a multi-layered experience that deepens cultural understanding beyond fragmented encounters.

### Cognitive-affective-behavioral model

In the cognitive-affective-behavioral model, rich cultural tourism experiences are associated with the cognitive aspect, as they involve the knowledge and understanding tourists gain about Ningxia’s cultural heritage [18]. Satisfaction represents the affective component, reflecting the emotional response tourists have after engaging with these experiences [10]. Finally, behavioral intention represents the behavioral aspect, indicating the likelihood that tourists will revisit or recommend Ningxia’s cultural heritage based on their experiences and satisfaction [19].

Tourists who have rich cultural tourism experiences in Ningxia tend to feel more satisfied with the region’s cultural heritage [20]. These immersive experiences deepen their appreciation and understanding, leading to greater overall contentment with their visit. Besides, tourists’ satisfaction with their cultural experiences in Ningxia significantly influences their behavioral intentions, such as the desire to revisit or recommend the destination to others [21]. When tourists feel content and emotionally fulfilled by their experience, they are more likely to form positive intentions, leading to actions that support and promote Ningxia’s cultural heritage in the future. It further extends the model by proposing that cognitive elements are defined not as generic experiences but as five distinct cultural dimensions (historical, folk, ecological, spiritual, modern) unique to heritage settings. The following hypotheses are presented:

- **Hypothesis (H1).** *Tourists’ rich cultural tourism experience has a positive influence on their satisfaction with Ningxia’s cultural heritage.*
- **Hypothesis (H2).** *Tourists’ satisfaction with Ningxia’s cultural heritage has a positive influence on their behavioral intention toward it.*

### Perceived cultural distance and cultural identity

Perceived Cultural Distance (PCD) moderates the links between Rich Cultural Tourism Experiences (RCTE), cultural identity, and satisfaction differently, based on specific cultural experience types [22,23]. When PCD is low, tourists feel more comfortable with local culture. This strengthens RCTE’s positive impact on cultural identity [22]. For example, a Han tourist joining Mid-Autumn Festival activities elsewhere easily connects with mooncake-gifting customs, boosting their shared identity but may not enhance their satisfaction. When PCD is high, effects vary: it weakens RCTE’s role in building identity for folk customs or rituals [23].But PCD doesn’t hurt satisfaction with historical sites (like a Southeast Asian tourist seeing the Great Wall) or exotic cuisines (like trying the lamb rice)-these rely on visuals or senses, not cultural alignment. It further extends the model by introducing two novel mechanisms: first, identifying cultural identity as a key mediator between the cognitive and behavioral components, a pathway underdeveloped in prior CAB research; second, it empirically tests how perceived cultural distance moderates these relationships in an ethnic minority context (Ningxia), clarifying boundary conditions of the framework-strengthening its explanatory power for culturally diverse tourism scenarios. Hence, the third hypothesis is proposed:

- **Hypothesis (H3a).** *Tourists’ perceived cultural distance negatively moderated the relationship between rich cultural tourism experience and cultural identity towards Ningxia’s cultural heritage.*
- **Hypothesis (H3b).** *Tourists’ perceived cultural distance negatively moderated the relationship between rich cultural tourism experience and cultural satisfaction towards Ningxia’s cultural heritage.*

Rich cultural tourism experiences significantly enhance cultural identity by facilitating direct engagement with both tangible and intangible aspects of culture. Activities such as participating in traditional tea ceremonies, attending local folk performances, or visiting historical monuments-like the ancient grottoes of Ningxia-provide immersive encounters that deepen tourists’ emotional and intellectual connections to the cultural context. Such concrete experiences help individuals internalize cultural symbols and narratives, thereby strengthening their sense of identity related to the destination [24]. Previous studies indicate that hands-on involvement, such as crafting local artifacts or learning indigenous cooking methods, leads to higher levels of cultural empathy and identity internalization [25].

This reinforced cultural identity contributes substantially to tourist satisfaction and behavioral intention. When visitors develop a meaningful attachment to the culture-through experiential activities such as ritual participation or heritage walks-they are more likely to perceive their travel experience as authentic and fulfilling [8]. Research shows that identified tourists exhibit greater tolerance toward situational inconveniences and report higher overall satisfaction, stemming from a sense of emotional belonging and symbolic value [26]. This strengthened cultural identity also fuels positive behavioral intentions: tourists with a deep attachment to Ningxia’s culture are more willing to revisit for further cultural exploration or recommend the destination to others, turning their satisfying experience into long-term engagement. The forth hypothesis is proposed:

**Hypothesis (H4).** *Tourists’ cultural identity positively influenced their satisfaction and behavioral intention towards Ningxia’s cultural heritage.*

Current cultural tourism research reveals two areas warranting further development: First, most studies use single-dimensional models, ignoring the complex interactions of multi-dimensional cultural experiences. Second, while perceived cultural distance is recognized, its inconsistent moderating effects on cultural identity and satisfaction in diverse destinations remain underexplored. This study addresses these by constructing a five-dimensional rich cultural tourism experience model and empirically testing the dual moderating role of perceived cultural distance, thus advancing theoretical understanding and practical strategies.

## Research methodology and data collection

### Research methodology

Study 1 utilizes content analysis, or text analysis, which entails an objective and systematic approach to describing the explicit content of communication or documentary materials [27]. This method is particularly effective for examining online texts related to cultural heritage reviews gathered through web scraping tools. During the content analysis process of Study 1, ROST CM 6 and MiniTag Cloud are used. ROST CM 6 (ROST Chinese Mining Toolkit Version 6) excels in large tourism text quantitative analysis-cutting segmentation errors, boosting domain-specific Chinese segmentation, and refining precision via its TF-IDF module. MiniTag Cloud’s (an online content analysis software: https://www.weiciyun.com) unique “thematic clustering with semantic proximity” algorithm excels at thematic extraction, grouping similar concepts into convergent themes with higher accuracy and conducting correlation analysis between two related aspects [1].In ROST CM6 software, pre-processing was performed by setting word segmentation options and filtering conditions such as stopword lists and part-of-speech screening to improve the accuracy of the analysis. First, the top 100 high-frequency words with a minimum frequency of 164 were identified and subjected to preliminary thematic clustering. Then, using MiniTag Cloud, words with a correlation coefficient exceeding 0.5 with “cultural experience” were summarized to extract the dimensions of “rich cultural motivations”.

To ensure the reliability and validity of rich cultural motivation dimensions, this study adopts the following dual steps. Reliability refers to the consistency of different coders in content categorization [28]. This study selected three master students with lexicography and research experience as coders. After training, they independently categorized 100 high-frequency words into groups by similar meanings. Holsti’s reliability coefficient was used to test consistency: pairwise consistent codings were 93, 95, 96 with agreement rates 0.93, 0.95, 0.96; average rate was 0.947, and Holsti’s coefficient 0.982 (>0.9), meeting reliability requirements [28]. For validity, classification accuracy was rechecked via original user reviews, and 3 tourism professionals independently reviewed results to ensure validity.

Study 2 involves 17 items spread across five constructs in the research model. Partial Least Squares Structural Equation Modeling (PLS-SEM) is particularly beneficial for predicting dependent variables and clarifying complex relationships [29]. As a multivariate statistical method, PLS-SEM is used to explore causal connections and structural models among variables. Compared to traditional SEM, PLS-SEM offers greater flexibility, making it well-suited for studies with smaller sample sizes. It does not require strict adherence to distributional assumptions and can simultaneously estimate both latent constructs and observed indicators. Using PLS-SEM will also be advantageous for evaluating reliability and validity [30].

### Sampling and data collection

Study 1 utilized the web scraping tool Octopus to collect reviews related to Ningxia’s cultural heritage from the C-trip travel app between April 2019 and June 2024. The data primarily included online reviews of seven famous cultural heritage sites in Ningxia: Helan Mountain Forest Park, Helan Mountain Rock Paintings, Water Cave Gully, Western Xia Mausoleum, Huangsha Ancient Ferry, Sand Lake, and Shapotou. A total of 14,163 relevant online texts were gathered.

Based on the results of the text analysis, Study 2 developed the questionnaire items. The questionnaire consists of 7 personal background questions and 17 questions related to tourists’ behavioral intentions (See S1 Table). Researchers collected 603 questionnaires from 14^th^ February 2024–5^th^ August 2024 through the online method. All questionnaire respondents were aged 18 or above. The first page of the survey served as an informed consent form, and participants were required to agree before proceeding to the formal questionnaire. This study utilized random sampling. Electronic questionnaires were distributed at Ningxia’s scenic spots entrances and on multiple social media platforms at the same time. For on-site surveys at Ningxia’s scenic spots, we used systematic random sampling by calculating intervals (1 in 10 visitors) via daily visitor counts, and used a random number generator for start points. Refusals were replaced only with the next interval visitor, ensuring equal selection chances. For online surveys, questionnaires were distributed via WeChat Moments, Weibo platform and diverse groups (e.g., travel discussion groups, regional community groups) by 4 research assistants. After completing the survey, respondents whose answers passed validity screening question received a 2 RMB red envelope as an incentive, which helped improve participation rate and data authenticity.

To ensure data validity, a cheating question asking “What is the name of Ningxia’s airport?” was included. Responses with incorrect or missing answers to this question were excluded from the analysis. After conducting checks for fraudulent responses and screening for response logic, the final number of valid questionnaires was 547, with a valid response rate of 90.71%.

This sample size aligns with PLS-SEM best practices, exceeding the 10-times rule for indicator variables. It matches typical tourism study ranges (400–600), ensuring statistical power and robust model estimation [31]. Therefore, the 547 valid samples are adequate for PLS-SEM, as PLS-SEM is robust for smaller samples (often≥100). The sample aligns with Ningxia’s broader tourist demographic: balanced gender ratio, dominant young adults (18–30), higher education levels, and diverse incomes mirror typical visitors, reflecting the region’s broad tourist appeal. The data collection and analysis methods strictly adhered to the terms and conditions of the data sources. This includes obtaining explicit consent for questionnaire respondents, anonymizing all personal information, and ensuring compliance with the data usage policies of online platforms from which public texts were collected. The dataset can be found in S1 Data for reference.

## Results

### Study 1

#### Analysis of the frequency of words in the reviews.

Word frequency analysis reveals the main content and structure of a text by calculating the frequency of each word’s occurrence. In this study, the occurrence of each word in the online texts was counted, and the top 100 high-frequency words were used to create a frequency chart to provide a clear understanding of tourists’ experiences after visiting Ningxia’s cultural heritage (see Table 1). Potential biases from web scraping-like missing reviews or skewed user content-were mitigated by cross-checking multiple platforms to ensure representativeness. Web scraping biases (missing reviews, skewed content) were mitigated via cross-checking. From April 2019 to June 2024, we used Octopus software to collect 14,162 reviews of Ningxia’s top 7 heritage spots on C-trip. Post-scraping, we cross-checked with Qunar website: filled missing Ctrip reviews and promotional ones via sentiment keywords like promotional keywords without genuine experience, ensuring data representativeness.

**Table 1:** High-Frequency Characteristic Words for Ningxia Cultural Heritage.

Word	Frequency	Word	Frequency	Word	Frequency	Word	Frequency
Scenic Area	4707	Attitude	561	Rich	300	Check-in	216
Worthwhile	3026	Environment	559	Sightseeing	296	Next Time	215
Project	2085	Excitement	538	National	292	Beautiful	210
Scenery	2058	Feelings	524	Weather	290	Can’t See	207
Service	1799	Interesting	519	Shocking	282	Sand Sliding	206
Rock Paintings	1650	Visit	513	Electric Car	282	Park	204
Desert	1630	Ruins	491	Performance	270	Going In	204
Helan Mountain	1457	Camel	472	Fun	269	Han Meilin	202
Staff	1415	Tools	468	Glass	255	Patience	200
History	1381	Tourists	466	Overall	254	Humanity	198
Location	1205	Suitable	466	Scenery	251	Archaeology	196
Museum	1131	Hours	464	Northwest	248	Knowledge	196
Western Xia	1089	Specialty	440	Child	244	Good Looking	190
Sand Lake	1083	Facilities	418	Minutes	243	Tomb	188
Explanation	1007	Guide	407	Then	240	Thanks	184
Landscape	956	Suggestions	400	Photo	239	Carriage	184
Yellow River	928	Great Desert	393	Route	237	Choice	175
Tour	899	Natural	361	Entire	232	Sand	175
Cost-Performance Ratio	841	Hygiene	346	Little Friend	231	Management	174
Enthusiasm	810	Touring	335	Spectacular	231	First Time	173
Playing	759	Intent	332	Super	228	Route	173
Ticket	724	Surfing	320	Considerate	227	Meaning	169
Convenient	709	Friends	313	Kilometers	226	Protection	166
Yinchuan	699	Western Xia	Mausoleum 312	Distance	224	Satisfaction	166
Culture	687	Art	308	Unique	221	Beautiful	164

Specifically, words like “history”, “museum”, “culture”, “Western Xia Mausoleum” and “archaeology” in the online reviews reflect the unique historical charm of Ningxia’s cultural heritage, representing the historical and cultural characteristics of these sites. Terms like “art” and “Han Meilin” highlight the artistic features of modern culture in Ningxia’s heritage, showing how the rich cultural foundation profoundly influences people’s perceptions. Words such as “camel”, “performance” and “sand sliding” reflect the folk culture of Ningxia’s heritage, illustrating the local cultural characteristics. “Desert”, “Helan Mountain”, “Sand Lake”, “Yellow River”, “ruins”, and “vast desert” are rooted in natural landscapes, showcasing the ecological culture of Ningxia’s heritage and highlighting the unique cultural heritage as a primary source of tourist perception. Furthermore, terms like “enthusiastic”, “convenient”, “attitude”, “exciting”, “interesting”, “distinctive”, “rich” and “fun” reveal tourists’ genuine feelings about their visits to Ningxia’s cultural heritage, which is an important reflection of the spiritual culture of these sites. From the analysis of the high-frequency words in Table 1, tourists’ experiences with Ningxia’s cultural heritage can be classified into five categories: historical, modern, folk, spiritual, and ecological experience.

The five experience categories are classified based on thematic clustering of high-frequency words: “history”, “Western Xia Mausoleum”, etc., focus on past heritage (historical experience); “art”, “Han Meilin” relate to contemporary creation (modern experience); “camel”, “performance” point to local customs (folk experience); “desert”, “Helan Mountain” rely on natural foundations (ecological experience); “enthusiastic”,“interesting” reflect emotional resonance (spiritual experience). Perceived cultural distance explains satisfaction inconsistencies: it weakens satisfaction in folk experience due to cultural differences but has little impact on universal experiences like historical and ecological ones.

**Fig 1:**
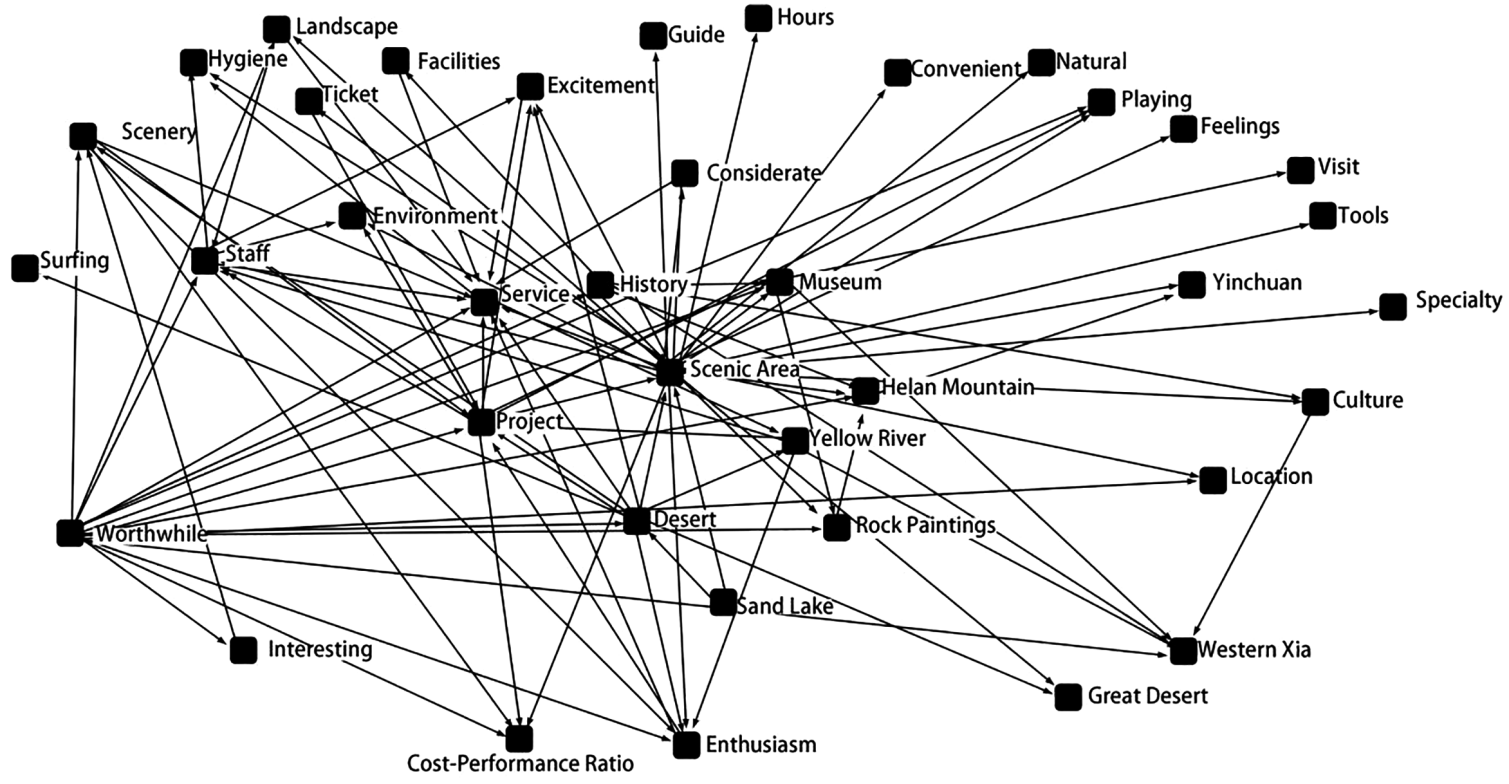
Semantic network diagram.

In the semantic network, nodes represent keywords, and edges represent the co-occurrence relationship between two keywords. If two keywords appear together in a segment of text, they are considered to have a co-occurrence relationship. The frequency of co-occurrence between keywords indicates the strength of the connections in the network; the more frequently keywords co-occur, the stronger their relationship. Fig 1 illustrates the density of connections based on the frequency of co-occurrence in the texts used by tourists to describe Ningxia’s cultural heritage. The greater the number of co-occurrences, the denser the lines in Fig 1, indicating a stronger association between the co-occurring words in tourists’ perceptions.

Overall, the network of keywords in the texts forms a semantic structure with “scenic area” as the primary center, and “Helan Mountain”, “Yellow River”, “Sand Lake”, “Museum” and “Desert” as secondary centers. The strong connections between “scenic area” and several high-frequency words like “Helan Mountain” ,“Yellow River”, “Sand Lake” and “Desert” indicate that cultural heritage is a key medium for tourists to further understand Ningxia. The strong association between “scenic area” and “Museum” suggests that museums play an increasingly important role in modern cultural tourism experiences. As globalization and cultural exchange accelerate, museums have become crucial places for tourists to learn about and experience different cultures. The strong connection between “scenic area” and “Helan Mountain”, “Yellow River” and “Desert” is directly related to Ningxia’s geographical environment. Helan Mountain and the Yellow River are significant natural landscapes in Ningxia, closely linked to the region’s ecological environment and cultural development, highlighting the impact of the ecological environment on cultural tourism experiences. Additionally, words like “rock paintings”, “Western Xia”, “history” and “culture” underscore Ningxia’s rich historical and cultural background. Terms such as “distinctive”, “exciting” and “enthusiastic” indicate the unique aspects of Ningxia’s cultural heritage and tourism experience, providing tourists with significant spiritual and cultural engagement. Words like “scenery”, “project” and “environment” reflect Ningxia’s folk culture, as they represent the region’s unique natural landscapes, traditional activities, and living conditions, allowing tourists to directly experience and appreciate Ningxia’s distinct local customs and way of life.

### Content analysis

Tung and Ritchie (2011) defined tourism experience as an individual’s subjective evaluation and the process they undergo, covering aspects of affect, cognition, and behavior [32]. Combined with the literature review of Hsu et al. (2010) and Chen & Rahman (2018), indicated that tourists’ cultural experiences are characterized by elements of “pleasant, experience, worthwhile and recommending” [25,33]. The present study sets out to develop an experience measurement method. It does so by performing a correlation analysis between the high-frequency words present in the online reviews of heritage and words associated with “pleasant, experience, worthwhile and recommending”, with the goal of identifying words related to cultural experience. These terms were selected as they align with Hsu et al. (2010) and Chen & Rahman (2018), capturing affect (pleasant), engagement (experience), value (worthwhile), and behavioral intent (recommending)-core facets of cultural experiences [25,33]. While other descriptors were considered, these four were prioritized for their established relevance and comprehensive coverage of key experiential dimensions. Online reviews of Ningxia’s heritage sites were collected and screened to ensure relevance. Using MiniTag Cloud software, the texts were processed through steps like word segmentation and stopword removal. High-frequency words were then extracted, followed by a correlation analysis between these words and the four characteristic elements with the Pearson correlation coefficient≥0.5. Through iterative coding and thematic clustering in the software, words strongly associated with cultural experience were identified, and five dimensions of rich cultural motivations were finally derived, as presented in Table 2.

**Table 2:** Development of measurable items.

Code	Measurable items	Related aspects	Correlation coefficient	Representative reviews
Historical experience	I would like to gain a rich understanding of its history and culture, including artifacts, **historical** records, sites, and ancient architecture of the cultural heritage site.	“Worthwhile” with “Historical”	0.501	“This place is rich in **historical** charm, making it a **worthwhile** destination for learning about the history of the Western Xia Dynasty.”
Modern experience	I would like to gain a rich understanding of its **modern** culture, including contemporary culture, art, distinctive performances, technological achievements, and cultural and creative products of the cultural heritage site.	“Worthwhile” with “Modern”	0.501	“The **modern** red-themed sand sculpture works, which are grand in scale, are really **worthwhile** for tourists to appreciate up close.”
Folk experience	I would like to gain a rich understanding of its **folk** culture, including local customs, lifestyle, cuisine, unique home-stays, and festivals of the cultural heritage site.	“Experience” with “Folk”	0.501	The **folk** Xixia – script printing technique is very popular among children. It is recommended that those with children go and **experience** this folk cultural activity.
Spiritual experience	I want to gain a gain a rich understanding of its **spiritual** culture, including interactions and integration into local life of the cultural heritage site.	“Pleasant” with “Spiritual”	0.502	“The staff were extremely kind and attentive. Their service made our visit not only **pleasant**, but also provided an incredibly wonderful experience at a **spiritual** level.”
Ecological experience	I want to gain a gain a rich understanding of its **ecological** culture, including natural landscapes and the ecological environment of the cultural heritage site.	“Recommending” with “Ecological”	0.502	“The **ecological** scenery is fascinating. The lavender manor is great, and the swans in the bird island are so beautiful. Small boats are shuttling through the reed marshes. It costs 80 yuan per person to ride a camel, and those who have never ridden one should give it a try. The experience is wonderful, the scenery is nice, and it’s definitely worth **recommending**.”

Each dimension holds unique value. The historical experience dimension, for instance, highlights the importance of heritage sites as repositories of history and culture. It implies that tourists are not just interested in the past but also see value in learning about it, which has implications for the preservation and interpretation of historical artifacts and sites. The modern experience dimension shows that contemporary culture and innovation are equally important in attracting tourists. Heritage sites can no longer rely solely on their historical charm but must also incorporate modern elements to remain relevant. The folk experience dimension emphasizes the role of local customs and traditions in creating a unique cultural experience. This dimension is particularly important for promoting cultural diversity and local identity. The spiritual experience dimension, on the other hand, underscores the importance of intangible aspects such as interactions and a sense of connection with the local community. The ecological experience dimension reveals the growing importance of nature-based tourism. As environmental awareness increases, heritage sites with beautiful natural landscapes can leverage this aspect to attract tourists.

Beyond correlation analysis, the study employed thematic association within MiniTag Cloud to explore how cultural experience words interact with perceptual and behavioral indicators. For instance, “historical” (from historical experience) frequently co-clustered with “worthwhile” (correlation coefficient = 0.501), directly shaping tourists’ cognitive evaluation that the site is valuable for learning, as seen in reviews emphasizing its historical charm as a reason for recommendation. Similarly, “folk” (folk experience) linked strongly with “experience” (correlation coefficient = 0.501), driving behavioral intentions like participating in Xixia-script printing activities, especially among families with children. Regarding perceived cultural distance, its influence is evident in the differential impact across dimensions. For folk experience, which relies on local customs, greater cultural distance may weaken the connection between “folk” and “experience”: tourists unfamiliar with Xixia culture might find the printing technique less engaging (correlation coefficient = 0.501). In contrast, historical experience (tied to universal historical value) and ecological experience (linked to “recommending” with 0.502) are less affected, as historical relics and natural landscapes have cross-cultural appeal.

Questionnaire items (see S1 Table) for each dimension are derived from text analysis: Historical experience (RCTE1) corresponds to frequently mentioned “cultural relics” and “ancient sites” reflecting the need to explore history. Modern experience (RCTE2) is based on terms like “cultural and creative products” and “technological achievements,” showing attention to contemporary elements. Folk experience (RCTE3) stems from “customs” and “cuisine” focusing on local traditions. Spiritual experience (RCTE4) comes from “interactions with locals” emphasizing immersive engagement. Ecological experience (RCTE5) is based on “natural landscapes” echoing environmental awareness. Items for cultural identity, satisfaction, behavioral intention, and perceived cultural distance are optimized by combining text themes with existing scales, accurately capturing tourists’ true feelings. Validation of questionnaire included expert reviews to assess content relevance, pilot testing with 50 tourists to refine clarity, and reliability checks (Cronbach’s α > 0.7) post-survey, confirming the dimensions accurately reflect rich cultural motivations.

### Study 2

#### Sample and data.

Gender distribution in this study is relatively balanced (48.99% male, 51.01% female), avoiding gender bias when analyzing experience-satisfaction links. Marital status reveals 57.22% unmarried participants (reflecting younger adults’ travel flexibility), echoed in 58.87% of visitors being 18–30 years old (see Table 3); in contrast, Ningxia’s overall Q1 2024 tourist age data cites 25–44-year-olds as the main group (62.90%) [34], indicating cultural heritage sites have stronger appeal for younger demographics (18–30). This youth dominance aligns with the CAB model’s “affective resonance” core: younger tourists’ pursuit of emotionally engaging experiences matches Ningxia’s RCTE design, driving their higher heritage engagement [2]. Income distribution (46.44% < 2,500 yuan; 32.36% 5,000–9,999 yuan) reflects accessibility across groups (see Table 3), which aligns with Ningxia’s Q1 2024 tourist occupation data-enterprise managers (26.10%), cultural/educational professionals (17.00%), service/sales staff (14.50%) are key visitor segments [34]. Lower-income tourists engage with free elements (basic cognitive input), while higher-income ones choose paid experiences (in-depth cognitive engagement)-directly reflecting the CAB model’s premise that experience depth shapes cognitive-affective-behavioral chains. Education-wise, 69.29% hold bachelor’s degrees or above (see Table 3), consistent with Qin (2021) [23] (higher education correlates with “deeper cultural engagement”), supporting our RCTE focus on historical and spiritual dimensions. Higher-educated tourists’ stronger cognitive understanding boosts satisfaction (cognitive → affective) and drives a 37.11% repeat visit rate (see Table 3). Ningxia’s Q1 2024 data notes 52.80% of domestic tourists are local [34], which may partially explain this relatively high repeat rate. Such repeat behavior stems from refined cultural cognition, realizing the CAB model’s affective → behavioral intention link.

**Table 3:** Descriptive Statistics.

Variable	n	%		n	%
**Gender**			**Education Level**		
Male	268	48.99	Primary School and Below	6	1.10
Female	279	51.01	Middle School	22	4.02
**Marital Status**			High School and Vocational School	74	13.53
Married	234	42.78	Higher Vocational College	35	6.40
Unmarried	313	57.22	Bachelor’s Degree	379	69.29
**Number of Visits**			Graduate Degree and Above	31	5.67
Once	146	26.69	**Average Monthly Income**		
2 Times	203	37.11	2500 RMB and below	254	46.44
3 Times	104	19.01	2500-4999 RMB	75	13.71
4 Times	44	8.04	5000-9999 RMB	177	32.36
4 Times and Above	50	9.14	10000-14999 RMB	30	5.48
**Age**			15000 RMB and above	11	2.01
18-30	322	58.87			
31-40	72	13.16			
41-50	77	14.08			
51-60	61	11.15			
61 and Above	15	2.74			

## Results

**Fig 2:**
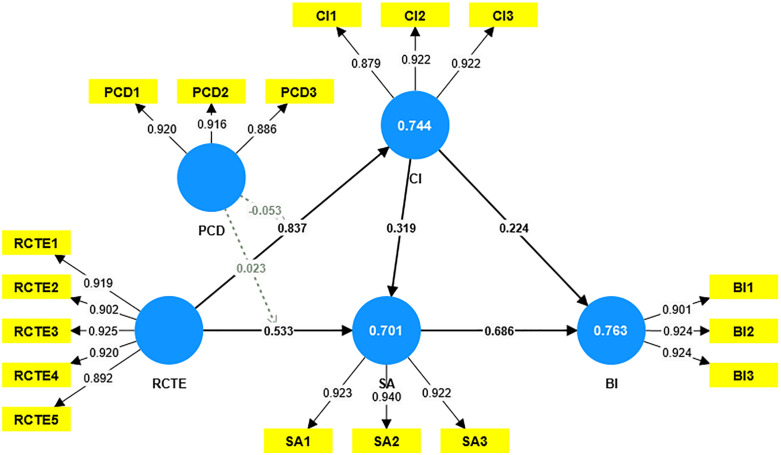
Results of model 1.

Fig 2 displays the results of the PLS-SEM (Partial Least Squares Structural Equation Modeling), where RCTE represents rich cultural tourism experience. PCD (perceived cultural distance) serves as a moderating variable in this model, while CI (cultural identity) acts as a mediating variable. Additionally, SA represents satisfaction, and BI indicates behavioral intention.

As shown in Fig 2 and Table 4, the model 1 results reveal that RCTE are crucial in enhancing cultural identity (CI) (path coefficient = 0.837, p-value = 0.000) and satisfaction (path coefficient = 0.053, p-value = 0.000), both of which positively influence tourists’ behavioral intentions. Therefore, Hypothesis 1 and 2 found to be accepted. Besides, CI significantly influences Behavioral Intention (BI) with a path coefficient of 0.224 (p-value: 0.000), implying that tourists who develop a strong cultural identity are more likely to have positive behavioral intentions, such as revisiting or recommending the destination [31]. In this sense, Hypothesis 4 was found to be supported. The three sub-dimensions of CI together play a significant positive mediating role between RCTE and BI, with each sub-dimension contributing distinctively. CI1 (“desire to immerse in local culture”) mediates by translating RCTE’s immersive qualities (e.g., participating in Ningxia’s folk rituals) into a proactive intent to integrate with the culture-this emotional and experiential drive bridges RCTE and subsequent BI. CI2 (“wish to engage with cultural elements”) mediates through targeted interaction: RCTE like the local embroidery workshops foster a desire to engage with specific cultural elements, which deepens psychological connection and fuels BI. CI3 (“aspire to gain cultural insight”) mediates via cognitive understanding: RCTE such as guided tours of Western Xia Mausoleum satisfy the desire for cultural insight, building a coherent cultural cognition that strengthens BI.

**Table 4:** Path Coefficients.

Path	Path Coefficients	Standard deviation	t-value	p-value	Hypothesis
**Model 1**					
RCTE - > SA	0.533	0.062	8.574	0.000	H1:accept
SA - > BI	0.686	0.055	12.517	0.000	H2:accept
PCD x RCTE - > CI	−0.053	0.021	2.546	0.011	H3a:accept
PCD x RCTE - > SA	0.023	0.031	0.733	0.463	H3b:reject
RCTE - > CI	0.837	0.022	38.139	0.000	H4:accept
CI - > BI	0.224	0.055	4.043	0.000	H4:accept
CI - > SA	0.319	0.059	5.364	0.000	
PCD - > CI	−0.001	0.024	0.050	0.960	
PCD - > SA	0.065	0.031	2.083	0.037	
**Model 2**					
RCTE1 - > SA	0.263	0.058	4.522	0.000	H1:accept
RCTE2 - > SA	0.075	0.055	1.377	0.169	
RCTE3 - > SA	0.090	0.061	1.473	0.141	
RCTE4 - > SA	0.229	0.055	4.135	0.000	
RCTE5 - > SA	0.242	0.058	4.179	0.000	
**Model 3**					
RCTE1 - > CI	0.244	0.052	4.684	0.000	H4:accept
RCTE2 - > CI	0.097	0.050	1.943	0.052	
RCTE3 - > CI	0.155	0.056	2.788	0.005	
RCTE4 - > CI	0.247	0.048	5.171	0.000	
RCTE5 - > CI	0.200	0.042	4.726	0.000	

While PCD does have some moderating effects, its impact is relatively minor compared to the direct effects of RCTE. Particularly, the interaction effect of PCD and RCTE on CI is negative and significant (path coefficient:-0.053, p-value: 0.011), suggesting that the relationship between rich cultural tourism experience and cultural identity is somewhat weakened when tourists perceive a greater cultural distance. However, the interaction effect of PCD and RCTE on SA is not significant (path coefficient: 0.023, p-value: 0.463), due to the perceived cultural distance does not meaningfully influence how a rich cultural tourism experience affects tourist satisfaction, suggesting that satisfaction is largely determined by the experience itself, regardless of cultural distance. In particular, PCD weakens RCTE’s impact on CI because larger cultural gaps hinder tourists’ cognitive and emotional alignment with local culture-for instance, unfamiliar local customs may reduce tourists’ sense of belonging, diluting identity formation. In contrast, PCD does not significantly affect RCTE’s influence on SA because satisfaction hinges more on tangible experience quality (e.g., immersive activities, service standards) than cultural alignment. Even with perceived distance, engaging cultural experiences directly drive contentment, making PCD irrelevant here. From this point of view, Hypothesis 3a was accepted but Hypothesis 3b was rejected.

The significant positive effects of Rich Cultural Tourism Experiences (RCTE) on Cultural Identity (CI) and Satisfaction (SA), which in turn drive Behavioral Intentions (BI), align closely with the Cognitive-Affective-Behavioral model. For instance, the path coefficient of 0.837 (p = 0.000) between RCTE and CI implies that destinations like Ningxia, by offering participatory activities such as the local embroidery workshops or guided tours of the Western Xia Mausoleum, can foster strong emotional and cognitive connections, directly translating into higher revisit and recommendation rates (e.g., a 1-unit increase in RCTE quality could potentially boost CI by 83.7%, subsequently increasing BI). However, the negative moderating effect of Perceived Cultural Distance (PCD) on the RCTE-CI relationship (path coefficient = −0.053, p = 0.011) introduces a critical managerial challenge: destinations must mitigate cultural barriers through strategies like cultural bridging (e.g., multilingual interpreters, pre-visit virtual reality experiences of the local rituals) to reduce cognitive dissonance for international tourists, as larger cultural gaps-may otherwise diminish identity formation by up to 5.3%. Conversely, the non-significant effect of PCD on satisfaction (path coefficient = 0.023, p = 0.463) underscores that satisfaction is driven primarily by tangible experience quality, meaning managers should prioritize consistent high-quality offerings, like well-trained guides in immersive workshops-regardless of cultural background, as these directly impact contentment and BI without cultural interference. Thus, real-world tourism management must adopt a dual approach: leveraging RCTE to enhance identity and satisfaction through culturally-rich, well-designed experiences, while simultaneously implementing targeted interventions to reduce cultural distance’s dampening effect on identity formation, ultimately optimizing long-term tourist engagement and destination loyalty.

**Fig 3:**
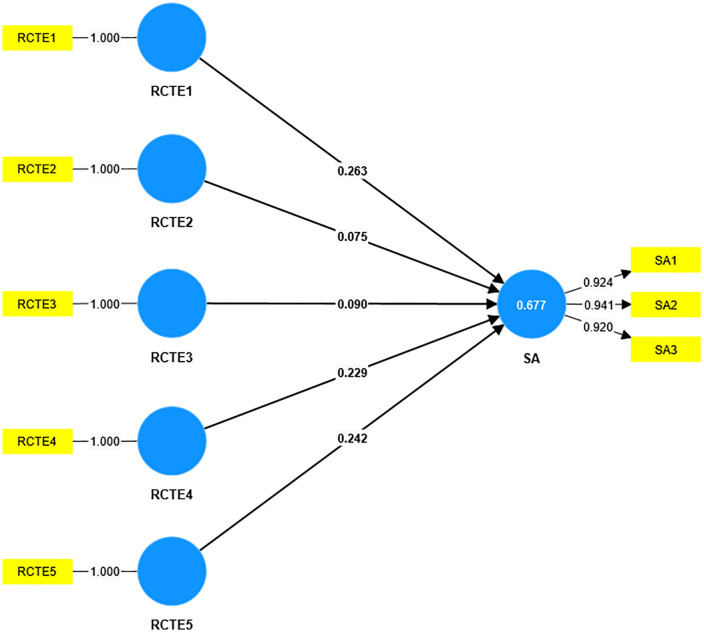
Results of model 2.

**Fig 4:**
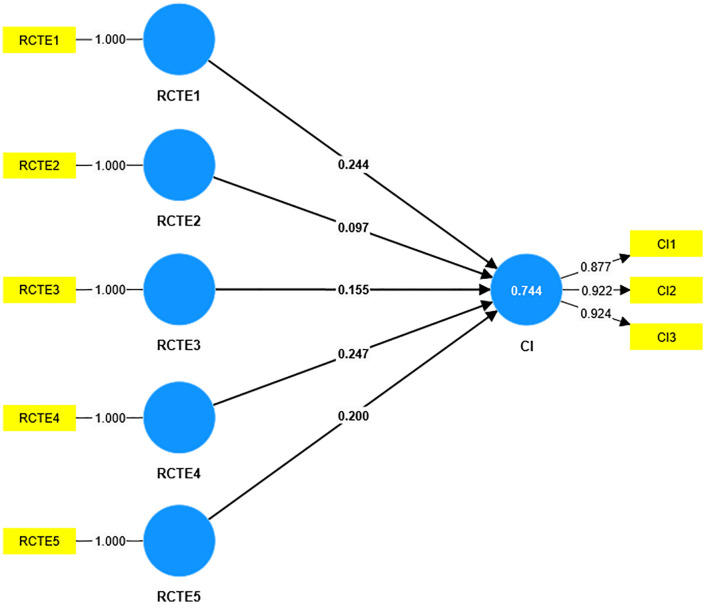
Results of model 3.

As shown in Fig 3 and Table 4, the model 2 results further elaborate that the historical, ecological, and spiritual aspects of rich cultural tourism experiences (RCTE) have significant stronger impacts on satisfaction because they align closely with the unique cultural and natural identity of Ningxia, offering tourists profound and memorable encounters. In contrast, modern and folk experiences may have a lesser impact as they either lack distinctiveness or fail to resonate as deeply with tourists’ expectations of cultural authenticity and connection. As shown in Fig 4 and Table 4, the model 3 results indicate that the spiritual, historical, and ecological aspects of-RCTE have a stronger impact on cultural identity because they evoke deeper emotional and intellectual connections to Ningxia’s heritage and natural environment, fostering a sense of belonging and appreciation. In contrast, modern and folk aspects may not resonate as strongly, as they are either less distinctive or fail to create the same level of cultural immersion and personal significance. Modern experiences may have weaker impacts because they often lean on generic cultural and creative products or standardized performances that lack uniqueness-failing to reflect Ningxia’s distinct regional characteristics, thus reducing immersion. Folk experiences might be perceived as less authentic due to commercialization: overly staged folk activities (e.g., simplified traditional rituals) can feel inauthentic, weakening tourists’ emotional connection to local culture.

Additionally, the mediating effect results presented in Table 5 indicate that cultural identity exerts a significant positive mediating influence on the relationship between RCTE and BI. PCD plays a significant negative moderation role between RCTE and CI, which plays an insignificant positive role between RCTE and SA.

**Table 5:** Results of mediating and moderating effect test.

Path	Effect Value	Standard deviation	t value	p-value
RCTE - > CI - > BI	0.188	0.047	3.998	0.000
PCD x RCTE - > CI - > BI	−0.012	0.005	2.202	0.028
PCD x RCTE - > SA - > BI	0.016	0.021	0.760	0.447

Table 6 presents the reliability and convergent validity results. Overall, in the measurement model, the suggested threshold for Composite Reliability (CR) should be greater than 0.7, and the internal consistency (Cronbach’s α) should also be greater than 0.7. Additionally, a rho value higher than 0.7 indicates that the composite construct has good reliability. The suggested threshold for the Average Variance Extracted (AVE) of the research constructs should be higher than 0.5 and factor loadings ≥ 0.7, indicating that the construct can explain more than 50% of the variance and each indicator strongly correlates with its respective construct [35,36]. All variables in this study’s model passed the reliability and validity tests, indicating that the measurement model in this study has good reliability.

**Table 6:** Reliability and Convergent Validity Results.

Variable	CA	rho	CR	AVE
BI	0.904	0.905	0.940	0.840
CI	0.894	0.896	0.934	0.825
PCD	0.893	0.906	0.933	0.823
RCTE	0.949	0.950	0.961	0.831
SA	0.920	0.921	0.949	0.862

Additionally, as illustrated in Table 7, the Cross-Loadings analysis for discriminant validity states that the loading of an indicator on its associated construct should be greater than all its loadings on other constructs. For instance, the loading of BI1 on BI is 0.901, which is greater than its cross-loadings on CI, PCD, RCTE and SA (0.728, 0.258, 0.764, and 0.821 respectively).

**Table 7:** Discriminant Validity Results (Cross-Loading Analysis).

	BI	CI	PCD	RCTE	SA
BI1	**0.901**	0.728	0.258	0.764	0.821
BI2	**0.924**	0.680	0.242	0.687	0.765
BI3	**0.924**	0.688	0.235	0.703	0.781
CI1	0.678	**0.879**	0.208	0.732	0.673
CI2	0.686	**0.922**	0.294	0.775	0.734
CI3	0.716	**0.922**	0.295	0.834	0.733
PCD1	0.251	0.299	**0.920**	0.336	0.326
PCD2	0.268	0.269	**0.916**	0.320	0.326
PCD3	0.203	0.224	**0.886**	0.274	0.263
RCTE1	0.713	0.800	0.303	**0.919**	0.766
RCTE2	0.709	0.757	0.300	**0.902**	0.716
RCTE3	0.696	0.790	0.332	**0.925**	0.742
RCTE4	0.741	0.802	0.336	**0.920**	0.762
RCTE5	0.717	0.772	0.298	**0.892**	0.747
SA1	0.769	0.704	0.321	0.742	**0.923**
SA2	0.819	0.760	0.335	0.792	**0.940**
SA3	0.812	0.724	0.289	0.747	**0.922**

## Discussion and implication

### Discussion

Study 1 identified five key dimensions of rich cultural tourism experience: historical, modern, folk, spiritual, and ecological cultural experience by using content analysis. These dimensions collectively represent the diverse ways tourists engage with a destination’s culture. By incorporating both tangible aspects, such as historical, modern and ecological experiences, and intangible elements, like spiritual and folk traditions, rich cultural tourism provides a multi-faceted engagement. This comprehensive approach ensures tourists can connect with a destination on multiple levels, fostering a more immersive and satisfying experience.

Study 2 found a significant positive relationship between rich cultural tourism experience and satisfaction, and between satisfaction and behavioral intention by using PLS-SEM. The rationale behind these findings lies in the depth and variety of experiences that rich cultural tourism offers. When tourists are deeply engaged with both the cultural past and the contemporary elements of a destination, their satisfaction levels increase. In the context of rich cultural tourism experiences, the historical, ecological, and spiritual dimensions demonstrate more substantial influences on both tourist satisfaction and the formation and perception of cultural identity, as opposed to the folk and modern dimensions. This heightened satisfaction is linked to stronger behavioral intentions, such as loyalty, revisits, and positive word-of-mouth. The more meaningful and enriching the experience, the more likely it is to convert into long-term engagement with the destination. Historical, ecological, and spiritual experiences resonate more likely because they align with core tourist expectations: historical aspects offer authentic heritage rooted in irreplaceable relics; ecological experiences provide unique natural landscapes inherent to Ningxia; spiritual dimensions foster genuine emotional connections via local interactions. In contrast, folk experiences may suffer from commercialization diluting authenticity, while modern ones often lack regional distinctiveness, failing to meet expectations for unique cultural immersion, thus weakening their impact.

Study 2 revealed cultural identity has been identified as an effective mediator between rich cultural tourism experience and behavioral intention. This is because the connection tourists form with the culture of a destination often leads to a stronger emotional attachment, fostering a sense of belonging. As tourists engage deeply with various dimensions of the local culture, they may experience an enhanced sense of cultural identity, which in turn strengthens their satisfaction and motivates positive behavioral intentions. Cultural identity in this study specifically refers to tourists’ emotional and cognitive connection with Ningxia’s regional culture (e.g., Western Xia heritage). Rich cultural tourism experiences strengthen tourists’ identification with Ningxia’s unique culture, which in turn boosts tourists’ behavioral intentions (revisits, recommendations). Therefore, enhancing Ningxia’s cultural tourism enriches tourists’ engagement with local culture, thereby strengthening tourists’ Ningxia-specific cultural identity. A reinforced cultural identity not only amplifies satisfaction but also solidifies the desire to revisit or promote the destination, making it a pivotal factor in sustaining tourist loyalty. Study 2 presented that perceived cultural distance indeed plays a moderating role, but its effects are inconsistent. While it significantly negatively moderates the relationship between rich cultural tourism experience and cultural identity, indicating that greater cultural differences may act as barriers to forming strong cultural bonds, its moderation of the relationship between rich cultural tourism experience and satisfaction is non-significant but positive. Perceived Cultural Distance (PCD) negatively moderates cultural identity not merely due to surface-level differences, but because it acts as a dual cognitive–emotional barrier that disrupts alignment with the target culture [23]. Cognitively, stark gaps in values, traditions, or norms (e.g., unfamiliar ritual logics or symbolic meanings) create confusion, making it hard for tourists to process and internalize cultural elements. Emotionally, this cognitive dissonance breeds alienation, dampening the sense of affinity needed to form a strong cultural connection. This suggests that although large cultural gaps may prevent tourists from fully identifying with a culture, they do not necessarily reduce their satisfaction. In fact, the novelty brought by cultural differences may even enhance the tourist experience in some cases, offering excitement and a fresh perspective, though it may not translate into richer cultural identification. This highlights the complex role perceived cultural distance plays in shaping different aspects of the tourism experience.

### Implication

From a theoretical perspective, this study makes several key contributions to the field of cultural tourism. First, it provides a refined definition of rich cultural tourism experience, emphasizing its multi-dimensional nature, including historical, modern, folk, spiritual, and ecological cultural experiences. The greater influence of historical, ecological, and spiritual dimensions (see model 2 and model 3 results in Table 4) on tourist satisfaction and cultural identity is because they tap into deeper human needs. History satisfies the longing for knowledge. Yang (2023) linked historical engagement to epistemic curiosity [13], and one tourist said, “Guide Mr.Chang explained Shuidonggou’s development, customs and history wittily. It’s great to listen to history while playing!”. Ecology provides a connection to nature, and spirituality offers inner fulfillment, while folk and modern aspects may not resonate as deeply, Ecology fosters a connection to nature. Nie et al. (2022) linked ecological tourism experiences to enhanced nature connectedness [4], and this is supported by a tourist’s feedback on Shapotou:“With mountains to the south, deserts to the north, and the Yellow River running through, this unique, magical natural scenery is utterly enchanting-an experience you can hardly get in cities, and even less so from ordinary folk cultural activities, which struggle to deliver such distinct, immersive connections.” For Ningxia, prioritize historical/ecological investments: enrich relic narratives and preserve landscapes. Improve folk experiences via authentic local participation (Salazar, 2012) and infuse modern elements with regional uniqueness to boost relevance, ensuring holistic cultural appeal [14]. The current study offers a fresh lens to understand tourist satisfaction and cultural identity, potentially guiding more targeted studies on tourism experiences and destination development. This holistic conceptualization deepens the understanding of how tourists engage with cultural destinations beyond superficial interactions, laying a stronger theoretical foundation for future research on cultural tourism. Second, by identifying and expanding on the critical dimensions of rich cultural tourism, the study extends the theoretical framework of rich cultural tourism and highlights its role in enhancing tourist satisfaction and behavioral intentions. Third, the research contributes to the literature by exploring the dual moderating effects of perceived cultural distance. It reveals the nuanced role that cultural distance plays in both hindering cultural identity formation and subtly enhancing satisfaction through novelty. Finally, this study enriches the application of the Cognitive-Affective-Behavioral (CAB) model within tourism management by demonstrating how cultural identity serves as an effective mediator between rich cultural tourism experience and behavioral intention, thereby extending the CAB mode’s relevance in explaining tourist behavior in cultural contexts.

This study advances the Cognitive-Affective-Behavioral (CAB) model in tourism management by clarifying its alignment-Cognitive corresponds to rich cultural tourism experiences (RCTE, covering historical, ecological, spiritual, modern, and folk dimensions), Affective to satisfaction (SA), and Behavioral to behavioral intention (BI)-while also identifying two key mechanisms: cultural identity (CI) as a mediator between RCTE and BI, and perceived cultural distance (PCD) as a significant negative moderator between RCTE and CI (though PCD exerts an insignificant positive effect between RCTE and SA). Practically, Ningxia’s tourism stakeholders can design targeted strategies to activate the CAB chain: For the historical dimension, deliver RCTE by using AR to restore scenes at Western Xia Mausoleum and pairing guides trained to simplify complex cultural contexts-this not only deepens historical understanding to boost SA but also reduces PCD, allowing RCTE to more effectively foster CI (which then drives BI); for the ecological dimension, organize RCTE like Yellow River eco-tours with sapling-planting, and add pre-tour materials explaining the ecological culture’s connection to local life-this enhances SA through nature connection and weakens PCD’s barrier to CI development; for the spiritual, modern, and folk dimensions, design RCTE such as Helan Mountain retreats with elder-led story sessions, vineyard tours blending grape-drying traditions with winemaking, and local-embroidery workshops, complemented by PCD-mitigating measures like cultural briefings on the local customs. These actions ensure RCTE smoothly converts to SA and effectively translates to CI, ultimately driving positive BI-helping stakeholders fully capitalize on RCTE’s value while addressing key contextual barriers.

Practical strategies can address these nuances: For re-visitors, design layered historical trails that unpack new narratives (e.g., linking Western Xia relics to contemporary folk traditions) and exclusive spiritual workshops with local elders, leveraging prior familiarity to deepen emotional resonance. For younger visitors, integrate AR technology into ecological tours to contextualize landscapes within ancient folklore and gamify authentic folk activities (e.g., interactive cooking classes using heirloom recipes), aligning with their digital-native engagement styles. For higher-educated tourists, offering scholarly programs such as archaeological field workshops or symposia on ecological sustainability and cultural adaptation, satisfying their desire for intellectual depth. To strengthen weaker dimensions, reimagine folk experiences by embedding them in daily life (e.g., participating in village harvest rituals) rather than staging them as spectacles, and infuse modern elements with regional symbolism (e.g., cultural products reinterpreting Helan Mountain rock art). Additionally, bridge cultural distance through educational interactions like guided discussions on the local customs, reducing identity barriers while preserving the novelty that sustains satisfaction. Such tailored approaches, grounded in demographic-specific engagement patterns, will deepen cultural identity and drive enduring behavioral intentions.

### Limitation and future research

First, the study’s sample focused on the Ningxia region, which may hinder the generalizability of results. Tourists’ perceptions of cultural distance and satisfaction can differ significantly across regions and cultural backgrounds, meaning findings may not apply to other cultural tourism contexts. Besides, the study focuses on domestic tourists, overlooking international visitors. Future studies can compare domestic and international tourists to explore cross-cultural identity dynamics and expand the sample to include diverse regions and cultural background is essential to validate the model’s generalizability. Additionally, this study exclusively used Chinese-language materials: Study 1 analyzed Chinese-only scenic spot reviews, and Study 2 employed Chinese questionnaires targeting domestic tourists. This language limitation, unaddressed in the manuscript, restricts cross-cultural generalizability. Future research should incorporate multilingual data to validate findings across diverse linguistic contexts. Cross-cultural comparative studies could explore how cultural distance moderates the relationship between rich cultural experiences, satisfaction, and behavioral intentions in different settings, while mixed-method approaches combining surveys with qualitative data could offer deeper insights into contextual nuances.

Second, this study uses cross-sectional data, which only provides a snapshot at a specific point in time and cannot capture the long-term changes in tourists’ experiences, satisfaction, cultural identification, and travel intentions related to cultural heritage. Since tourism experiences and cultural identification may change over time, cross-sectional data may not fully reflect these dynamic processes. Future research could adopt longitudinal data or follow-up study designs to explore the long-term relationships and trends between these variables more deeply. For instance, conducting longitudinal surveys over a 1–2 year period, ensuring coverage of both peak and off-tourism seasons to capture seasonal variations in visitor composition. The current analysis focuses primarily on the overall direct relationship between cultural identity and behavioral intention, future studies can incorporate demographic variables (age, income) as moderators in the model. Additionally, this study does not compare first-time and repeat visitors-for example, examing whether CI correlates more strongly with BI or if CI subdimensions’ mediating roles differ across the two groups. This comparison is proposed in future research, which will distinguish visitor types and use appropriate statistical tests to explore such differences.

Finally, the study relies on self-reported questionnaire data, which may introduce self-report bias or social desirability bias. The measurement of variables such as tourist experiences, satisfaction, cultural identification, and travel intentions may be influenced by individual subjective perceptions and response tendencies, potentially affecting the accuracy and reliability of the data. The self-reported data may bring common method bias. We adopted measures like inserting the cheating question and using clear wording. Future research could incorporate other data collection methods, such as interviews, observations, or behavioral data analysis, to validate and supplement the results of self-reported data, providing a more comprehensive research perspective.

## Supporting information

S1 Table Measurement scales. (DOCX)

S1 Data Questionnaire Results. (CSV)
