# Supplementary material for: Impact of rich cultural tourism experience on tourist satisfactions and behavioral intentions toward Ningxia’s cultural heritage: Moderation role of perceived cultural distance
Source: PLoS One. 2025 Nov 6;20(11):e0336220. doi: 10.1371/journal.pone.0336220 (PMC12591403; doi:10.1371/journal.pone.0336220)
Supplement: S1 Table — (DOCX) [file pone.0336220.s001.docx]

**S1 Table. Measurement scales.**

| Code | Measurable items |
| --- | --- |
|  | **Historical cultural experience** |
| RCTE1 | I would like to gain a rich understanding of its history and culture, including artifacts, historical records, sites, and ancient architecture of the cultural heritage site. |
|  | **Modern cultural experience** |
| RCTE2 | I would like to gain a rich understanding of its modern culture, including contemporary culture, art, distinctive performances, technological achievements, and cultural and creative products of the cultural heritage site. |
|  | **Folk cultural experience** |
| RCTE3 | I would like to gain a rich understanding of its folk culture, including local customs, lifestyle, cuisine, unique home-stays, and festivals of the cultural heritage site. |
|  | **Spiritual culture experience** |
| RCTE4 | I want to gain a gain a rich understanding of its spiritual culture, including interactions and integration into local life of the cultural heritage site. |
|  | **Ecological cultural experience** |
| RCTE5 | I want to gain a gain a rich understanding of its ecological culture, including natural landscapes and the ecological environment of the cultural heritage site. |
|  | **Cultural identity** |
| CI1 | I desire to immerse myself in the local culture in the cultural heritage destination. |
| CI2 | I wish to engage with the cultural elements of the cultural heritage. |
| CI3 | I aspire to gain insight into the local culture in the cultural heritage. |
|  | **Satisfaction** |
| SA1 | Compared to my expectations, my experience at this cultural heritage is good. |
| SA2 | Overall, I am very pleased with this cultural heritage. |
| SA3 | In comparison to other cultural heritages, I am extremely satisfied with this one |
|  | **Behavioral intention** |
| BI1 | I intend to return to this cultural heritage if I visit the area again. |
| BI2 | I would like to come back to this cultural heritage in five years. |
| BI3 | Whenever I get the chance, I would visit this cultural heritage as my first choice. |
|  | **Perceived cultural distance** |
| PCD1 | I find the customs and traditions at the cultural heritage destination to be distinctly different from cultural heritage in my hometown. |
| PCD2 | I perceive a notable difference in the manner of communication at the cultural heritage destination compared to cultural heritage in my hometown. |
| PCD3 | I feel a significant cultural disparity between the cultural heritage destination and cultural heritage in my hometown. |
